# Correction: Diagnostic performance of congestion score index evaluated from chest radiography for acute heart failure in the emergency department: A retrospective analysis from the PARADISE cohort

**DOI:** 10.1371/journal.pmed.1003562

**Published:** 2021-03-05

**Authors:** Masatake Kobayashi, Amine Douair, Kevin Duarte, Déborah Jaeger, Gaetan Giacomin, Adrien Bassand, Victor Jeangeorges, Laure Abensur Vuillaume, Gregoire Preud’homme, Olivier Huttin, Faiez Zannad, Patrick Rossignol, Tahar Chouihed, Nicolas Girerd

The 8^th^ author’s name was entered into the submission system incorrectly and therefore was indexed incorrectly on PubMed. The correct name is Laure Abensur Vuillaume. The correct indexing in PubMed should read Abensur Vuillaume L.
