# Supplementary material for: Diagnostic performance of congestion score index evaluated from chest radiography for acute heart failure in the emergency department: A retrospective analysis from the PARADISE cohort
Source: PLoS Med. 2020 Nov 11;17(11):e1003419. doi: 10.1371/journal.pmed.1003419 (PMC7657510; doi:10.1371/journal.pmed.1003419)
Supplement: S1 Checklist — (DOCX) [file pmed.1003419.s001.docx]

**S1 Checklist. TRIPOD checklist**

| **Section/Topic Item Checklist Item Page** | | | |
| --- | --- | --- | --- |
| **Title and abstract** |  |  |  |
| **Title** | 1 | Identify the study as developing and/or validating a multivariable prediction model, the target population, and the outcome to be predicted. | Title page |
| **Abstract** | 2 | Provide a summary of objectives, study design, setting, participants, sample size, predictors, outcome, statistical analysis, results, and conclusions. | Abstract section |
| **Introduction** |  |  |  |
| **Background and objectives** | 3a | Explain the medical context (including whether diagnostic or prognostic) and rationale for developing or validating the multivariable prediction model, including references to existing models. | Introduction section  Paragraph 1-3 |
|  | 3b | Specify the objectives, including whether the study describes the development or validation of the model or both. | Introduction section Paragraph 4 |
| **Methods** |  |  |  |
| **Source of data** | 4a | Describe the study design or source of data (e.g., randomized trial, cohort, or registry data), separately for the development and validation data sets, if applicable. | Study population (Methods)  Paragraph 1 |
|  | 4b | Specify the key study dates, including start of accrual; end of accrual; and, if applicable, end of follow-up. | Study population (Methods)  Paragraph 1 |
| **Participants** | 5a | Specify key elements of the study setting (e.g., primary care, secondary care, general population) including number and location of centers. | Study population (Methods) Paragraph 1 |
|  | 5b | Describe eligibility criteria for participants. | Study population (Methods) Paragraph 1 |
|  | 5c | Give details of treatments received, if relevant. | — |
| **Outcome** | 6a | Clearly define the outcome that is predicted by the prediction model, including how and when assessed. | Diagnosis of Heart Failure (Methods) Paragraph 1 |
|  | 6b | Report any actions to blind assessment of the outcome to be predicted. | Diagnosis of Heart Failure (Methods) Paragraph 1 |
| **Predictors** | 7a | Clearly define all predictors used in developing or validating the multivariable prediction model, including how and when they were measured. | Radiographic Congestion Score Index (Methods)  Paragraph 1 |
|  | 7b | Report any actions to blind assessment of predictors for the outcome and other predictors. | Radiographic Congestion Score Index (Methods)  Paragraph 2 |
| **Sample size** | 8 | Explain how the study size was arrived at. | Statistical analysis (Methods)  Paragraph 3 |
| **Missing data** | 9 | Describe how missing data were handled (e.g., complete-case analysis, single imputation, multiple imputation) with details of any imputation method. | Statistical analysis (Methods)  Paragraph 5 |
| **Statistical analysis methods** | 10a | Describe how predictors were handled in the analyses. | Statistical analysis (Methods)  Paragraph 3 |
|  | 10b | Specify type of model, all model-building procedures (including any predictor selection), and method for internal validation. | Statistical analysis (Methods)  Paragraph 3 |
|  | 10d | Specify all measures used to assess model performance and, if relevant, to compare multiple models. | Statistical analysis (Methods)  Paragraph 3 |
| **Risk groups** | 11 | Provide details on how risk groups were created, if done. | — |
| **Results** | | | |
| **Participants** | 13a | Describe the flow of participants through the study, including the number of participants with and without the outcome and, if applicable, a summary of the follow-up time. A diagram may be helpful. | S1 Fig |
|  | 13b | Describe the characteristics of the participants (basic demographics, clinical features, available predictors), including the number of participants with missing data for predictors and outcome. | Baseline Characteristics (Results) Paragraph 1 and 2 & S1 Fig |
| **Model development** | 14a | Specify the number of participants and outcome events in each analysis. | Association of Congestion Score Index with Adjudicated Discharge Diagnosis of Acute Heart Failure (Results) Paragraph 1 |
|  | 14b | If done, report the unadjusted association between each candidate predictor and outcome. | Association of Congestion Score Index with Adjudicated Discharge Diagnosis of Acute Heart Failure (Results) Paragraph 1 |
| **Model specification** | 15a | Present the full prediction model to allow predictions for individuals (i.e., all regression coefficients, and model intercept or baseline survival at a given time point). | Diagnostic Value of the Congestion Score Index (Results) Paragraph 1 and 2 |
|  | 15b | Explain how to the use the prediction model. | Diagnostic Value of the Congestion Score Index (Results) Paragraph 1 and 2 |
| **Model performance** | 16 | Report performance measures (with CIs) for the prediction model. | Diagnostic Value of the Congestion Score Index (Results) Paragraph 1 and 2 |
| **Discussion** |  |  |  |
| **Limitations** | 18 | Discuss any limitations of the study (such as nonrepresentative sample, few events per predictor, missing data). | Limitations and strengths (Discussion) Paragraph 1- 4 |
| **Interpretation** | 19b | Give an overall interpretation of the results, considering objectives, limitations, and results from similar studies, and other relevant evidence. | Discussion  Paragraph 1- 5 |
| **Implications** | 20 | Discuss the potential clinical use of the model and implications for future research. | Discussion Paragraph 1  (Clinical implications -Paragraph 1) |
| **Other information** |  |  |  |
| **Supplementary information** | 21 | Provide information about the availability of supplementary resources, such as study protocol, Web calculator, and data sets. | — |
| **Funding** | 22 | Give the source of funding and the role of the funders for the present study. | Funding |
