# Supplementary material for: Diagnostic performance of congestion score index evaluated from chest radiography for acute heart failure in the emergency department: A retrospective analysis from the PARADISE cohort
Source: PLoS Med. 2020 Nov 11;17(11):e1003419. doi: 10.1371/journal.pmed.1003419 (PMC7657510; doi:10.1371/journal.pmed.1003419)
Supplement: S1 Fig — (DOCX) [file pmed.1003419.s002.docx]

**S1 Fig. Flow Chart**


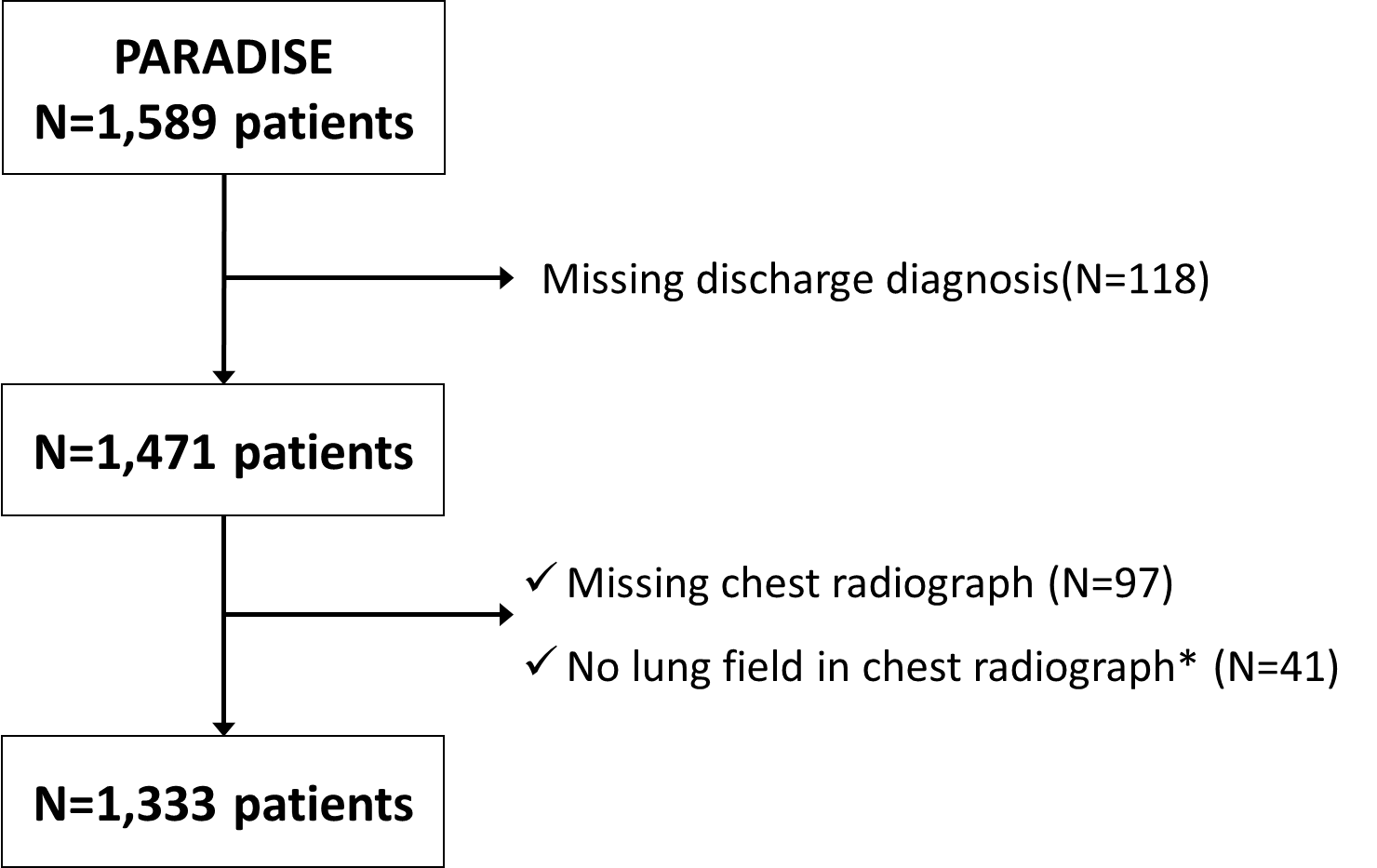
*Patients had no available lung field in their chest radiograph due to pleural effusion, atelectasis or cardiac silhouette.
