# Supplementary material for: Diagnostic performance of congestion score index evaluated from chest radiography for acute heart failure in the emergency department: A retrospective analysis from the PARADISE cohort
Source: PLoS Med. 2020 Nov 11;17(11):e1003419. doi: 10.1371/journal.pmed.1003419 (PMC7657510; doi:10.1371/journal.pmed.1003419)
Supplement: S1 Table — (DOCX) [file pmed.1003419.s003.docx]

**S1 Table. Baseline Characteristics of Patients with Available and Unavailable Chest Radiograph**

|  | Global  (N=1,471) | Chest radiograph  Unavailable (N=138) | Chest radiograph  Available (N=1,333) | p-value |
| --- | --- | --- | --- | --- |
| Age, yrs | 70.1 ± 20.1 | 51.4 ± 24.7 | 72.0 ± 18.5 | **<0.0001** |
| Men, N (%) | 750 (51.0 %) | 66 (47.8 %) | 684 (51.3 %) | 0.44 |
| Body mass index, kg/m² | 25.5 ± 5.5 | 25.2 ± 4.8 | 25.5 ± 5.5 | 0.80 |
| Medical history, N (%) |  |  |  |  |
| Hypertension | 773 (52.5 %) | 44 (31.9 %) | 729 (54.7 %) | **<0.0001** |
| Diabetes mellitus | 318 (21.6 %) | 16 (11.6 %) | 302 (22.7 %) | **0.003** |
| Dyslipidemia | 298 (20.3 %) | 18 (13.0 %) | 280 (21.0 %) | **0.027** |
| Coronary artery disease | 176 (12.0 %) | 14 (10.1 %) | 162 (12.2 %) | 0.49 |
| Atrial fibrillation | 327 (22.2 %) | 14 (10.1 %) | 313 (23.5 %) | **0.0003** |
| Heart failure | 275 (18.7 %) | 17 (12.3 %) | 258 (19.4 %) | **0.044** |
| Prior HF admission | 95 (7.1 %) | NA | 95 (7.1 %) | NA |
| Medication, N (%) |  |  |  |  |
| ACEi/ARB | 468 (33.1 %) | 24 (18.0 %) | 444 (34.7 %) | **0.0001** |
| Beta-blocker | 323 (22.9 %) | 17 (12.8 %) | 306 (23.9 %) | **0.004** |
| Spironolactone | 70 (5.0 %) | 6 (4.5 %) | 64 (5.0 %) | 0.80 |
| Diuretics | 387 (27.4 %) | 19 (14.3 %) | 368 (28.8 %) | **0.0004** |
| Calcium channel blocker | 276 (19.5 %) | 18 (13.5 %) | 258 (20.2 %) | 0.066 |
| Statin | 323 (22.9 %) | 23 (17.3 %) | 300 (23.5 %) | 0.11 |
| O2 flow, L/min | 4.0 (2.0 - 9.0) | 6.0 (3.0 - 9.0) | 4.0 (2.0 - 9.0) | **0.024** |
| Physical examination, N (%) |  |  |  |  |
| Leg edema | 345 (23.5 %) | 11 (8.0 %) | 334 (25.1 %) | **<0.0001** |
| Jugular venous distension | 45 (3.1 %) | 2 (1.5 %) | 43 (3.3 %) | 0.38 |
| Rales | 454 (35.2 %) | NA | 454 (35.2 %) | NA |
| Systolic BP, mmHg | 131.8 ± 25.7 | 128.8 ± 22.0 | 132.1 ± 26.0 | 0.17 |
| Diastolic BP, mmHg | 73.7 ± 17.4 | 75.9 ± 14.9 | 73.5 ± 17.6 | **0.032** |
| Heart rate, bpm | 95.6 ± 20.7 | 95.0 ± 20.8 | 95.7 ± 20.7 | 0.71 |
| Respiratory rate, /min | 26.2 ± 8.0 | 24.3 ± 8.9 | 26.3 ± 7.9 | **0.004** |
| Laboratory findings |  |  |  |  |
| Hemoglobin, g/dl | 12.8 ± 2.0 | 12.6 ± 2.1 | 12.8 ± 2.0 | 0.73 |
| White blood count | 11200  (8200 - 15400) | 10100  (8100 - 14000) | 11300  (8300 - 15400) | 0.20 |
| C-reactive protein, mg/dl | 65.8 (17.8 - 140.0) | 42.0 (10.5 - 102.4) | 66.4 (18.0 - 140.0) | 0.18 |
| Sodium, mmol/l | 137.0 ± 5.4 | 138.6 ± 8.0 | 136.9 ± 5.3 | 0.32 |
| Potassium, mmol/l | 4.1 ± 0.6 | 4.1 ± 0.6 | 4.1 ± 0.6 | 0.92 |
| Blood glucose, mmol/l | 7.7 ± 3.5 | 7.5 ± 3.2 | 7.7 ± 3.5 | 0.78 |
| BUN, mg/dl | 25.5 ± 19.2 | 28.9 ± 38.0 | 25.3 ± 18.0 | 0.43 |
| eGFR, ml/min/1.73m² | 82.5 ± 54.9 | 79.9 ± 37.0 | 82.6 ± 55.4 | 0.75 |
| BNP, pg/ml | 269 (125 - 586) | 115 (59 - 280) | 274 (133 - 590) | **0.011** |
| Blood gas |  |  |  |  |
| PH | 7.41 (7.34 - 7.45) | 7.40 (7.31 - 7.46) | 7.41 (7.34 - 7.45) | 0.94 |
| PaO2, mmHg | 65.0 (56.0 - 79.0) | 65.0 (52.0 - 80.5) | 65.0 (56.0 - 79.0) | 0.81 |
| PaCO2, mmHg | 40.0 (35.0 - 48.0) | 39.5 (32.5 - 47.5) | 40.4 (35.0 - 48.0) | 0.46 |
| Lactate, mmol/L | 1.10 (0.80 - 1.60) | 1.05 (0.75 - 1.55) | 1.10 (0.80 - 1.60) | 0.64 |
| Diagnosis of AHF at discharge | 300 (20.4 %) | 11 (8.0 %) | 289 (21.7 %) | **0.0001** |

Values are mean ±SD, n (%) or median (25th to 75th percentile)

HF, heart failure; ACEi, angiotensin converting enzyme inhibitor; ARB, angiotensin receptor blocker; BP, blood pressure; BUN, blood urea nitrogen; eGFR, estimated glomerular filtration rate; BNP, brain natriuretic peptide; PH, potential of hydrogen ;PaO2, pa*r*tial pressure of oxygen; PaCO2, partial pressure of carbon dioxide; AHF, acute heart failure.
