# Supplementary material for: Diagnostic performance of congestion score index evaluated from chest radiography for acute heart failure in the emergency department: A retrospective analysis from the PARADISE cohort
Source: PLoS Med. 2020 Nov 11;17(11):e1003419. doi: 10.1371/journal.pmed.1003419 (PMC7657510; doi:10.1371/journal.pmed.1003419)
Supplement: S2 Table — (DOCX) [file pmed.1003419.s004.docx]

**S2 Table. Patient Characteristics across Different Discharge Diagnoses**

|  | Acute heart failure (N=289) | | | | non acute heart failure (N=1044) | | |  |
| --- | --- | --- | --- | --- | --- | --- | --- | --- |
|  | **ACS (N=12)** | **Lung disease**  **(N=189)** | **AF (N=8)** | **Others (N=80)** | **COPD/**  **Asthma (N=286)** | **Bronchitis/**  **Pneumonia (N=541)** | **Others (N=217)** | **p-value** |
| Age, yrs | 82.0 ± 9.7 | 82.7 ± 9.3 | 77.6 ± 11.3 | 80.6 ± 11.9 | 67.5 ± 17.4 | 73.2 ± 18.0 | 61.9 ± 22.3 | <0.001 |
| Men, N (%) | 6 (50.0 %) | 90 (47.6 %) | 6 (75.0 %) | 36 (45.0 %) | 150 (52.4 %) | 271 (50.1 %) | 127 (58.5 %) | 0.17 |
| Body mass index, kg/m² | 25.3 ± 5.1 | 27.1 ± 6.8 | 29.1 ± 4.0 | 26.3 ± 5.6 | 25.6 ± 5.9 | 25.2 ± 5.0 | 24.4 ± 4.7 | <0.001 |
| Medical history, N (%) |  |  |  |  |  |  |  |  |
| Hypertension | 10 (83.3 %) | 141 (74.6 %) | 4 (50.0 %) | 56 (70.0 %) | 136 (47.6 %) | 295 (54.5 %) | 87 (40.1 %) | <0.001 |
| Diabetes mellitus | 5 (41.7 %) | 71 (37.6 %) | 4 (50.0 %) | 29 (36.2 %) | 57 (19.9 %) | 106 (19.6 %) | 30 (13.8 %) | <0.001 |
| Coronary artery disease | 5 (41.7 %) | 36 (19.0 %) | 3 (37.5 %) | 19 (23.8 %) | 41 (14.3 %) | 46 (8.5 %) | 12 (5.5 %) | <0.001 |
| Atrial fibrillation | 5 (41.7 %) | 83 (43.9 %) | 4 (50.0 %) | 30 (37.5 %) | 48 (16.8 %) | 114 (21.1 %) | 29 (13.4 %) | <0.001 |
| Heart failure | 5 (41.7 %) | 86 (45.5 %) | 5 (62.5 %) | 42 (52.5 %) | 39 (13.6 %) | 61 (11.3 %) | 20 (9.2 %) | <0.001 |
| Prior HF admission, N (%) | 2 (16.7 %) | 37 (19.6 %) | 4 (50.0 %) | 22 (27.5 %) | 11 (3.8 %) | 13 (2.4 %) | 6 (2.8 %) | <0.001 |
| Medication, N (%) |  |  |  |  |  |  |  |  |
| ACEi/ARB | 7 (63.6 %) | 80 (43.7 %) | 3 (37.5 %) | 40 (51.3 %) | 85 (30.8 %) | 179 (34.8 %) | 50 (24.0 %) | <0.001 |
| Beta-blocker | 4 (36.4 %) | 63 (34.4 %) | 6 (75.0 %) | 34 (43.6 %) | 39 (14.1 %) | 123 (23.9 %) | 37 (17.8 %) | <0.001 |
| Diuretics | 3 (27.3 %) | 84 (45.9 %) | 4 (50.0 %) | 35 (44.9 %) | 80 (29.0 %) | 135 (26.2 %) | 27 (13.0 %) | <0.001 |
| Statin | 8 (72.7 %) | 55 (30.1 %) | 4 (50.0 %) | 24 (30.8 %) | 67 (24.3 %) | 108 (21.0 %) | 34 (16.3 %) | <0.001 |
| O2 flow, L/min (N=627) | 9.0 (3.0 - 12.0) | 4.0 (3.0 - 9.0) | 6.0 (3.0 - 9.0) | 6.0 (2.0 - 9.0) | 3.0 (2.0 - 6.0) | 4.0 (2.0 - 9.0) | 6.0 (2.5 - 9.0) | 0.063 |
| Physical examination, N (%) |  |  |  |  |  |  |  |  |
| Leg edema | 6 (50.0 %) | 108 (57.1 %) | 4 (50.0 %) | 45 (56.2 %) | 58 (20.3 %) | 86 (15.9 %) | 27 (12.4 %) | <0.001 |
| Jugular venous distension | 3 (27.3 %) | 13 (7.0 %) | 0 (0.0 %) | 7 (9.0 %) | 4 (1.4 %) | 11 (2.1 %) | 5 (2.3 %) | <0.001 |
| Rales | 6 (50.0 %) | 124 (67.8 %) | 5 (83.3 %) | 45 (59.2 %) | 75 (27.0 %) | 158 (30.4 %) | 41 (19.2 %) | <0.001 |
| Systolic BP, mmHg | 130.0 ± 26.8 | 139.1 ± 27.7 | 131.0 ± 26.3 | 138.3 ± 31.9 | 132.5 ± 23.6 | 128.3 ± 25.9 | 132.9 ± 24.0 | <0.001 |
| Heart rate, bpm | 104.8 ± 21.7 | 92.1 ± 20.6 | 101.1 ± 21.0 | 92.1 ± 23.2 | 96.8 ± 19.4 | 97.7 ± 20.6 | 92.9 ± 21.0 | 0.001 |
| Respiratory rate, /min | 26.3 ± 9.8 | 28.3 ± 8.2 | 23.2 ± 4.4 | 26.0 ± 7.2 | 25.8 ± 6.8 | 26.6 ± 8.3 | 24.7 ± 8.4 | 0.006 |
| Congestion score index | 2.2 ± 0.5 | 2.1 ± 0.6 | 2.5 ± 0.4 | 2.2 ± 0.6 | 1.1 ± 0.7 | 1.4 ± 0.7 | 0.9 ± 0.7 | <0.001 |
| Laboratory findings |  |  |  |  |  |  |  |  |
| Hemoglobin, g/dl | 13.2 ± 1.8 | 12.4 ± 1.8 | 12.7 ± 3.5 | 12.1 ± 2.0 | 13.5 ± 1.8 | 12.6 ± 2.0 | 13.0 ± 2.1 | <0.001 |
| White blood count, µ/l | 14118  (11300 - 18005) | 13212  (9100 - 16470) | 15540  (10140 - 19540) | 12203  (7800 - 15180) | 11905  (8420 - 14300) | 13005  (8000 - 16200) | 11348  (8100 - 13600) | 0.005 |
| C-reactive protein, mg/dl | 3.5 (1.0 - 7.1) | 7.8 (2.6 - 13.8) | 2.0 (1.1 - 5.2) | 2.2 (1.0 - 6.5) | 3.0 (1.0 - 9.1) | 10.2 (4.3 - 17.6) | 2.4 (0.8 - 11.2) | <0.001 |
| Sodium, mmol/l | 136.9 ± 3.4 | 137.5 ± 4.3 | 138.1 ± 4.6 | 136.3 ± 5.8 | 136.6 ± 4.6 | 136.8 ± 6.1 | 137.2 ± 4.5 | 0.026 |
| Potassium, mmol/l | 4.2 ± 0.6 | 4.2 ± 0.7 | 4.3 ± 0.4 | 4.4 ± 0.6 | 4.2 ± 0.5 | 4.1 ± 0.6 | 4.1 ± 0.5 | <0.001 |
| Blood glucose, mmol/l | 12.6 ± 4.5 | 8.9 ± 3.9 | 12.4 ± 10.3 | 9.3 ± 4.5 | 7.0 ± 2.5 | 7.6 ± 3.3 | 7.0 ± 2.8 | <0.001 |
| BUN, mg/dl | 27.1 ± 12.1 | 30.3 ± 17.8 | 30.5 ± 15.7 | 33.9 ± 26.4 | 19.9 ± 14.3 | 26.7 ± 19.0 | 20.8 ± 12.1 | <0.001 |
| eGFR, ml/min/1.73m² | 71.0 ± 26.9 | 65.6 ± 28.5 | 57.8 ± 24.0 | 63.2 ± 36.8 | 91.9 ± 36.9 | 84.7 ± 74.6 | 89.0 ± 35.3 | <0.001 |

Values are mean ±SD, n (%), median (25th to 75th percentile) or mean (25th to 75th percentile) in white blood cell count

ACS, acute coronary syndrome; AF, atrial fibrillation; COPD, chronic obstructive pulmonary disease; HF, heart failure; ACEi, angiotensin converting enzyme inhibitor; ARB, angiotensin receptor blocker; BP, blood pressure; BUN, blood urea nitrogen; eGFR, estimated glomerular filtration rate.
