# Supplementary material for: Diagnostic performance of congestion score index evaluated from chest radiography for acute heart failure in the emergency department: A retrospective analysis from the PARADISE cohort
Source: PLoS Med. 2020 Nov 11;17(11):e1003419. doi: 10.1371/journal.pmed.1003419 (PMC7657510; doi:10.1371/journal.pmed.1003419)
Supplement: Attachment — Submitted filename: CSI_PARADISE_responses_to_reviewers.docx [file pmed.1003419.s005.docx]

**Ref.: Ms. No. PMEDICINE-D-20-02033R1**

**Diagnostic Performance of Congestion Score Index Evaluated from Chest Radiography for Acute Heart Failure in the Emergency Department**

**PLOS Medicine**

**Requests from the editors:
*Please revise your title according to PLOS Medicine's style - ideally this should have the study design in a subtitle (after the main study question is summarized) after a colon - eg, "A randomized controlled trial," "A retrospective study," "A modelling study," etc.**

We have modified our title according to PLOS Medicine’s style. Thank you.

“Diagnostic Performance of Congestion Score Index Evaluated from Chest Radiography for Acute Heart Failure in the Emergency Department: An analysis from the PARADISE retrospective cohort.”

***We'd ask the authors restructure the abstract using the PLOS Medicine headings (Background, Methods and Findings, Conclusions) - "methods and findings" should be a single subsection.
*In the last sentence of the Abstract Methods and Findings section, please describe the main limitation(s) of the study's methodology.**
According to these instructions, we have modified our abstract as below. Thank you.

“**Methods and Findings:** The diagnostic value of CSI for acute HF (adjudicated from patients’ discharge files) was studied in the PARADISE cohort (Pathway of dyspneic patients in Emergency). CSI was evaluated using a semi-quantitative method on chest radiography in consecutive patients admitted for acute dyspnea in the emergency department. Results were validated in independent cohorts (N=224). Of 1,333 patients, mean (SD) age was 72.0 (18.5) years, 686 (51.5%) were men, and mean (SD) CSI was 1.42 (0.79). Patients with higher CSI had more cardiovascular comorbidities, more severe congestion, higher b-type natriuretic peptide (BNP), poorer renal function and more respiratory acidosis. Acute HF was diagnosed in 289 (21.7%) patients. CSI was significantly associated with acute HF diagnosis (adjusted-odds ratio for 0.1-unit CSI increase 1.19, 95%CI 1.16 to 1.22). The diagnostic accuracy of CSI for acute HF was excellent, whether alone [area under ROC curve (AUROC) 0.84, 95%CI 0.82 to 0.86] or in addition to clinical model (AUCROC 0.87, 95%CI 0.85 to 0.90). CSI improved diagnostic accuracy on top of clinical variables [Net reclassification improvement (NRI)=94.9%] and clinical variables plus BNP (NRI=55.0%). Good diagnostic accuracy was observed in the validation cohorts (AUROC 0.75, 95%CI 0.68 to 0.82). The key limitation of our derivation cohort was its single-center and retrospective nature, which was counterbalanced by the validation in the independent cohorts.”

***At this stage, we ask that you include a short, non-technical Author Summary of your research to make findings accessible to a wide audience that includes both scientists and non-scientists. The Author Summary should immediately follow the Abstract in your revised manuscript. This text is subject to editorial change and should be distinct from the scientific abstract. Please see our author guidelines for more information:** [**https://journals.plos.org/plosmedicine/s/revising-your-manuscript#loc-author-summary**](https://journals.plos.org/plosmedicine/s/revising-your-manuscript#loc-author-summary)
We have incorporated the following “author summary” in our revised manuscript.

**Author summary**

**Why was this study done?**

• Chest radiography is often performed in patients admitted for acute dyspnea in the emergency department but its assessment is generally not standardized.

• Latest guidelines for heart failure emphasize the limitations of chest radiography in its current use.

• A standardized approach to quantify pulmonary congestion from chest radiography might improve its diagnostic performance for acute heart failure.

**What did the researchers do and find?**

• We studied the diagnostic value of a semi-quantitative approach (the Congestion Score Index) to pulmonary congestion on chest radiography for acute heart failure in a retrospective cohort study of 1,333 patients with acute dyspnea admitted to the emergency department.

• This Congestion Score Index was significantly and strongly associated with acute heart failure diagnosis.

• The Congestion Score Index also improved diagnostic accuracy on top of clinical parameters, and on top of clinical parameters and natriuretic peptide.

• This good diagnostic accuracy of the Congestion Score Index was externally validated in independent cohorts (N=224).

**What do these findings mean?**

• The quantification of radiographic pulmonary congestion using the Congestion Score Index improved diagnostic accuracy for acute heart failure on top of clinical parameters and natriuretic peptide.

***Ideally, please reformat the in-text citations to use sequential numerals in square brackets (eg [1], [2] etc) rather than superscript numerals - if using referencing software this should be relatively straight forward.**
We have corrected in-text citations per this instruction. Thank you.

***Did your study have a prospective protocol or analysis plan? Please state this (either way) early in the Methods section.**

**a) If a prospective analysis plan (from your funding proposal, IRB or other ethics committee submission, study protocol, or other planning document written before analyzing the data) was used in designing the study, please include the relevant prospectively written document with your revised manuscript as a Supporting Information file to be published alongside your study, and cite it in the Methods section. A legend for this file should be included at the end of your manuscript.
b) If no such document exists, please make sure that the Methods section transparently describes when analyses were planned, and when/why any data-driven changes to analyses took place.
c) In either case, changes in the analysis-- including those made in response to peer review comments-- should be identified as such in the Methods section of the paper, with rationale.**
The current study is a prospective analysis of a retrospective cohort. However, we do not have a written analysis plan. We have added this important point in the method section of the revised manuscript as below:

“This analysis on the PARADISE cohort was planned in February 2019 planning for the MD thesis of AD. TC and NG planned to evaluate the diagnostic value of CSI for AHF in the ED; however, no formal analysis was written at the time. Of note, among all patients with available data, chest radiography was evaluated blinded for clinical data and diagnosis, and standard statistical approached were used.”

As you can see, a number of features prevent data-driven changes. The statistical analysis is standard approach as outlined by the statistician reviewer.

***The authors could consider using a reporting guideline designed to help enhance reporting of diagnostic studies, eg either STARD (**[**https://www.equator-network.org/reporting-guidelines/stard/**](https://www.equator-network.org/reporting-guidelines/stard/)**) or TRIPOD (**[**https://www.equator-network.org/reporting-guidelines/tripod-statement/**](https://www.equator-network.org/reporting-guidelines/tripod-statement/)**) depending on which is more appropriate to this study. If using one of the guidelines please complete and upload the appropriate checklist as supporting information alongside the revised paper (and consider citing the paper for the guideline in the methods section, noting that the tool was used to guide reporting of your study)**

| **Section/Topic Item Checklist Item Page** | | | |
| --- | --- | --- | --- |
| **Title and abstract** |  |  |  |
| **Title** | 1 | Identify the study as developing and/or validating a multivariable prediction model, the target population, and the outcome to be predicted. | Title P1 |
| **Abstract** | 2 | Provide a summary of objectives, study design, setting, participants, sample size, predictors, outcome, statistical analysis, results, and conclusions. | Abstract section P2 |
| **Introduction** |  |  |  |
|  |  | Explain the medical context (including whether diagnostic or prognostic) and rationale for developing or validating the multivariable prediction model, including references to existing models. | Introduction section P3 |
| **Background and objectives** | 3a |  |  |
|  | 3b | Specify the objectives, including whether the study describes the development or validation of the model or both. | Introduction P3 L22-25 |
| **Methods** |  |  |  |
| **Source of data** | 4a | Describe the study design or source of data (e.g., randomized trial, cohort, or registry data), separately for the development and validation data sets, if applicable. | Study population (Methods) P4 L3 |
|  | 4b | Specify the key study dates, including start of accrual; end of accrual; and, if applicable, end of follow-up. | Study population (Methods) P4 L5 |
| **Participants** | 5a | Specify key elements of the study setting (e.g., primary care, secondary care, general population) including number and location of centers. | Study population (Methods) P4 L4-5 |
|  | 5b | Describe eligibility criteria for participants. | Study population (Methods) P4 L5-8 |
|  | 5c | Give details of treatments received, if relevant. | — |
| **Outcome** | 6a | Clearly define the outcome that is predicted by the prediction model, including how and when assessed. | Diagnosis of Heart Failure (Methods) P5 L2-8 |
|  | 6b | Report any actions to blind assessment of the outcome to be predicted. | Diagnosis of Heart Failure (Methods) P5 L2-8 |
| **Predictors** | 7a | Clearly define all predictors used in developing or validating the multivariable prediction model, including how and when they were measured. | Radiographic Congestion Score Index (Methods)  P5 L10 - P6 L2 |
|  | 7b | Report any actions to blind assessment of predictors for the outcome and other predictors. | Radiographic Congestion Score Index (Methods)  P5 L24-25 |
| **Sample size** | 8 | Explain how the study size was arrived at. | Statistical analysis (Methods)  P6 L19-20 |
| **Missing data** | 9 | Describe how missing data were handled (e.g., complete-case analysis, single imputation, multiple imputation) with details of any imputation method. | Statistical analysis (Methods)  P7 L6-7 |
| **Statistical analysis methods** | 10a | Describe how predictors were handled in the analyses. | Statistical analysis (Methods)  P6 L10- P7 L4 |
|  | 10b | Specify type of model, all model-building procedures (including any predictor selection), and method for internal validation. | Statistical analysis (Methods)  P6 L10- P7 L4 |
|  | 10d | Specify all measures used to assess model performance and, if relevant, to compare multiple models. | Statistical analysis (Methods)  P6 L10- P7 L4 |
| **Risk groups** | 11 | Provide details on how risk groups were created, if done. | — |
| **Results** | | | |
| **Participants** | 13a | Describe the flow of participants through the study, including the number of participants with and without the outcome and, if applicable, a summary of the follow-up time. A diagram may be helpful. | Supplementary figure 1 |
|  | 13b | Describe the characteristics of the participants (basic demographics, clinical features, available predictors), including the number of participants with missing data for predictors and outcome. | Baseline Characteristics (Results) P7 L0-16 & Supplementary figure 1 |
| **Model development** | 14a | Specify the number of participants and outcome events in each analysis. | Results P7 L22 |
|  | 14b | If done, report the unadjusted association between each candidate predictor and outcome. | Results P7 L22-24 |
| **Model specification** | 15a | Present the full prediction model to allow predictions for individuals (i.e., all regression coefficients, and model intercept or baseline survival at a given time point). | Results P8 L2-13 |
|  | 15b | Explain how to the use the prediction model. | Results P8 L2-13 |
| **Model performance** | 16 | Report performance measures (with CIs) for the prediction model. | Results P8 L2-13 |
| **Discussion** |  |  |  |
| **Limitations** | 18 | Discuss any limitations of the study (such as nonrepresentative sample, few events per predictor, missing data). | Discussion Limitations and strengths P12 |
| **Interpretation** | 19b | Give an overall interpretation of the results, considering objectives, limitations, and results from similar studies, and other relevant evidence. | Discussion P9-11 |
| **Implications** | 20 | Discuss the potential clinical use of the model and implications for future research. | Discussion clinical implications P11 |
| **Other information** |  |  |  |
| **Supplementary information** | 21 | Provide information about the availability of supplementary resources, such as study protocol, Web calculator, and data sets. | — |
| **Funding** | 22 | Give the source of funding and the role of the funders for the present study. | Acknowledgements P13 |

**Reviewer #1:**

We thank this reviewer for his/her detailed feedback and constructive comments.

The reviewers’ comments are stated in plain text **with bold letters**, the authors’ answers in plain text.

**Reviewer #1: Authors reported and concluded that semi-quantified assessment of pulmonary congestion on chest radiography provided excellent diagnostic value for acute heart failure in dyspneic patients in the emergency settings. Furthermore, this assessment may be available method for acute heart failure triage in the emergency department. This finding is interesting, however, I have the following concerns.**

**1. Vascular congestion is the most obvious therapeutic target when approaching patients with acute decompensated heart failure. From the view point of evaluation methods to identify congestion, cardiac imaging including chest X-ray is highly needed. However, initial screening to diagnose congestion or acute heart failure is mainly performed by physical examinations in clinical practice; natriuretic peptides is not routinely measured.**
Thank you for this thoughtful comment of our results. We agree that natriuretic peptides are not always measured in routine clinical practice. In the latest clinical recommendations, when AHF diagnosis is unequivocal based on clinical examination, treatment should begin rather than waiting for further testing in acute onset dyspneic patients. However, in these guidelines, the measurement of natriuretic peptide (BNP, NT-proBNP or MR-proANP) is also recommended in all patients with acute dyspnea and suspected AHF to help in the differentiation of AHF from non-cardiac causes of acute dyspnea. In this regard, the present study showed that CSI improved diagnostic accuracy on top of the Brest score (based on clinical history, physical examination and electrocardiogram [1]), and on top of the Brest score plus BNP. Importantly, our results did suggest that CSI provided the improvement of diagnostic performance on top of clinical-based model. Per this reviewer suggestion, we have emphasized that AHF diagnosis is generally determined from clinical parameters in our revised manuscript as below.

Discussion section entitled “**Radiographic Congestion Score Index and Other Diagnostic Measurements: Clinical Parameters and Natriuretic Peptide**”

“In the latest guidelines, natriuretic peptide is recommended to rule out non-HF related causes of acute dyspnea, although accumulated data suggested the overall diagnostic accuracy of natriuretic peptide [2-6]. However, multiple comorbidity burden (i.e. older age and poor renal function) may lead to diagnostic uncertainly in a sizeable proportion of dyspneic patients[7]. BNP, in addition, requires time to measure and is not always available in routine clinical practice. In cases with unequivocal diagnosis of AHF based on clinical parameters, prompt treatment approach is recommended rather than wait for its result [8]. In this regard, the Brest score, based on clinical parameters, may be pragmatic tool as well as good diagnostic accuracy for diagnosing HF as previously shown [1].”

(references)

1.	Basset A, Nowak E, Castellant P, Gut-Gobert C, Le Gal G, L'Her E. Development of a clinical prediction score for congestive heart failure diagnosis in the emergency care setting: The Brest score. Am J Emerg Med. 2016;34(12):2277-83. Epub 2016/09/08. doi: 10.1016/j.ajem.2016.08.023. PubMed PMID: 27599400.

2.	Krauser DG, Chen AA, Tung R, Anwaruddin S, Baggish AL, Januzzi JL, Jr. Neither race nor gender influences the usefulness of amino-terminal pro-brain natriuretic peptide testing in dyspneic subjects: a ProBNP Investigation of Dyspnea in the Emergency Department (PRIDE) substudy. J Card Fail. 2006;12(6):452-7. Epub 2006/08/17. doi: 10.1016/j.cardfail.2006.04.005. PubMed PMID: 16911912.

3.	Anwaruddin S, Lloyd-Jones DM, Baggish A, Chen A, Krauser D, Tung R, et al. Renal function, congestive heart failure, and amino-terminal pro-brain natriuretic peptide measurement: results from the ProBNP Investigation of Dyspnea in the Emergency Department (PRIDE) Study. J Am Coll Cardiol. 2006;47(1):91-7. Epub 2006/01/03. doi: 10.1016/j.jacc.2005.08.051. PubMed PMID: 16386670.

4.	Bayes-Genis A, Lloyd-Jones DM, van Kimmenade RR, Lainchbury JG, Richards AM, Ordonez-Llanos J, et al. Effect of body mass index on diagnostic and prognostic usefulness of amino-terminal pro-brain natriuretic peptide in patients with acute dyspnea. Arch Intern Med. 2007;167(4):400-7. Epub 2007/02/28. doi: 10.1001/archinte.167.4.400. PubMed PMID: 17325303.

5.	Januzzi JL, Jr., Chen-Tournoux AA, Christenson RH, Doros G, Hollander JE, Levy PD, et al. N-Terminal Pro-B-Type Natriuretic Peptide in the Emergency Department: The ICON-RELOADED Study. J Am Coll Cardiol. 2018;71(11):1191-200. Epub 2018/03/17. doi: 10.1016/j.jacc.2018.01.021. PubMed PMID: 29544601.

6.	Daniels LB, Maisel AS. Natriuretic peptides. J Am Coll Cardiol. 2007;50(25):2357-68. doi: 10.1016/j.jacc.2007.09.021. PubMed PMID: 18154959.

7.	Roberts E, Ludman AJ, Dworzynski K, Al-Mohammad A, Cowie MR, McMurray JJ, et al. The diagnostic accuracy of the natriuretic peptides in heart failure: systematic review and diagnostic meta-analysis in the acute care setting. BMJ (Clinical research ed). 2015;350:h910. Epub 2015/03/06. doi: 10.1136/bmj.h910. PubMed PMID: 25740799; PubMed Central PMCID: PMCPMC4353288.

8.	Mebazaa A, Yilmaz MB, Levy P, Ponikowski P, Peacock WF, Laribi S, et al. Recommendations on pre-hospital & early hospital management of acute heart failure: a consensus paper from the Heart Failure Association of the European Society of Cardiology, the European Society of Emergency Medicine and the Society of Academic Emergency Medicine. Eur J Heart Fail. 2015;17(6):544-58. Epub 2015/05/23. doi: 10.1002/ejhf.289. PubMed PMID: 25999021.

**2. In this study, the signs and symptoms, including jugular venous distention, rales, and leg edema, are relatively low even in patients among Quartile IV. Although the authors indicated the accuracy of radiographic congestion index for acute heart failure, it may be unreasonable to conclude the utility of this index for acute heart failure triage in the emergency department.**
Thank you for this detailed feedback. In the current study, a relatively low number of patients (21.7%) were diagnosed AHF at discharge, which may explain the fact that a minority of patients had congestion signs (i.e., leg edema, rales and jugular venous distention). Importantly, in our center (as in most centers in France), some patients known to have HF were directly admitted in the intensive cardiac care unit/cardiology ward, not through the emergency department [1]. Therefore, this low proportion of AHF diagnosis possibly due to French health care system may influence our results. However, it should be noted that, recently, hospitalization rates for worsening HF in the ED declined over past decades [2]. It may result from the development of disease management program to prevent urgent HF hospitalization [3]. Indeed, this proportion of AHF diagnosis and frequencies of congestion signs were similar to those in dyspneic patients in contemporary data [4, 5]. However, we agree that these relatively low frequencies of congestion signs are considered as a limitation in the present study and have added this point in our revised manuscript as follows:

Limitations

“The overall proportion of AHF diagnosis was relatively low, which may explain the fact that a low number of patients had congestion signs (i.e., leg edema, rales and jugular venous distention) and cardiovascular diseases. In our center (as in most centers in France), some dyspneic patients known to have HF and all patients with obvious evidence of myocardial ischemia were admitted directly in the intensive cardiac care unit/cardiology ward, not through the ED [1]. The proportion of AHF diagnosis possibly due to the health care system may influence our results. However, it should be noted that hospitalization rates for worsening HF in the ED declined over past decades [2], which may result from the development of disease management program to prevent urgent HF hospitalization [3]. Indeed, the proportion of AHF diagnosis in the present study was similar to that of dyspneic patients in other contemporary cohorts [4, 5].”

(references)

1.	Logeart D, Isnard R, Resche-Rigon M, Seronde MF, de Groote P, Jondeau G, et al. Current aspects of the spectrum of acute heart failure syndromes in a real-life setting: the OFICA study. Eur J Heart Fail. 2013;15(4):465-76. Epub 2012/11/29. doi: 10.1093/eurjhf/hfs189. PubMed PMID: 23186936.

2.	Schmidt M, Ulrichsen SP, Pedersen L, Bøtker HE, Sørensen HT. Thirty-year trends in heart failure hospitalization and mortality rates and the prognostic impact of co-morbidity: a Danish nationwide cohort study. Eur J Heart Fail. 2016;18(5):490-9. Epub 2016/02/13. doi: 10.1002/ejhf.486. PubMed PMID: 26868921.

3.	Bradley EH, Curry L, Horwitz LI, Sipsma H, Wang Y, Walsh MN, et al. Hospital strategies associated with 30-day readmission rates for patients with heart failure. Circulation Cardiovascular quality and outcomes. 2013;6(4):444-50. Epub 2013/07/19. doi: 10.1161/circoutcomes.111.000101. PubMed PMID: 23861483; PubMed Central PMCID: PMCPMC3802532.

4.	Januzzi JL, Jr., Chen-Tournoux AA, Christenson RH, Doros G, Hollander JE, Levy PD, et al. N-Terminal Pro-B-Type Natriuretic Peptide in the Emergency Department: The ICON-RELOADED Study. J Am Coll Cardiol. 2018;71(11):1191-200. Epub 2018/03/17. doi: 10.1016/j.jacc.2018.01.021. PubMed PMID: 29544601.

5.	Ibrahim I, Kuan WS, Frampton C, Troughton R, Liew OW, Chong JP, et al. Superior performance of N-terminal pro brain natriuretic peptide for diagnosis of acute decompensated heart failure in an Asian compared with a Western setting. Eur J Heart Fail. 2017;19(2):209-17. Epub 2016/09/14. doi: 10.1002/ejhf.612. PubMed PMID: 27620387.

**3. Generally, the presence of pneumonia may obscure the appearance or degree of congestion in patients with decompensated heart failure. In this paper, the mean value of C-reactive protein was 6.6 mg/dL in global population, and no description of patients with clinically apparent pneumonia in the excluding criteria. And serum procalcitonin level could be useful to handle such patients, affecting key results.**

In the PARADISE cohort, investigators systematically reviewed the hospital’s electronic charts (Resurgences®) and found the records of all patients admitted for acute dyspnea in the emergency department (even if they had pneumonia or chronic obstructive pulmonary disease). Below, we present additional descriptive table according to all discharge diagnosis (i.e. not just AHF versus no-AHF). Indeed, in patients with dyspnea precipitated by bronchitis/pneumonia, median C-reactive protein was 10.2 (4.3-17.6) mg/dl, which was consistent with that reported in a previous report [1]. For the sake of clarity, we did not describe this additional table in the revised version; however, if deemed necessary by this reviewer and/or the editorial team, we would show this additional descriptive table.

We truly agree that pneumonia is often perceived as a limitation to analyzed pulmonary congestion on chest radiography. However, importantly, as emphasized in our forest plot (**Figure 3**) of the revised manuscript, the diagnostic accuracy of CSI is not affected by the presence of pneumonia diagnosis at discharge [C-index(95%CI)= 0.79 (0.76-0.83) in patients with pneumonia and 0.89 (0.86-0.93) in those without pneumonia]. This result strongly suggested the usefulness of CSI even in the setting of coexisting pneumonia. We better acknowledge this important point in the revised version of the manuscript. Thank you for this important comment which further strengthen our manuscript.

Discussion section entitled “**Radiographic Congestion Score Index as a Diagnostic Tool for Acute Heart Failure**

“However, our subgroup analysis provided a remarkably homogenous diagnostic performance of CSI across various subgroups (i.e., elderly, body mass index) and coexisting lung diseases (i.e., COPD and pneumonia).”

(reference)

1.	Le Bel J, Hausfater P, Chenevier-Gobeaux C, Blanc FX, Benjoar M, Ficko C, et al. Diagnostic accuracy of C-reactive protein and procalcitonin in suspected community-acquired pneumonia adults visiting emergency department and having a systematic thoracic CT scan. Critical care (London, England). 2015;19:366. Epub 2015/10/17. doi: 10.1186/s13054-015-1083-6. PubMed PMID: 26472401; PubMed Central PMCID: PMCPMC4608327.

**Patient Characteristics across Different Discharge Diagnoses**

|  | Acute heart failure | | | | non acute heart failure | | |  |
| --- | --- | --- | --- | --- | --- | --- | --- | --- |
|  | **ACS (N=12)** | **Lung disease (N=189)** | **AF (N=8)** | **Others (N=80)** | **COPD/**  **Asthma (N=286)** | **Bronchitis/**  **Pneumonia (N=514)** | **Others (N=217)** | **p-value** |
| Age, yrs | 82.0 ± 9.7 | 82.7 ± 9.3 | 77.6 ± 11.3 | 80.6 ± 11.9 | 67.5 ± 17.4 | 73.2 ± 18.0 | 61.9 ± 22.3 | <0.001 |
| Men, N (%) | 6 (50.0 %) | 90 (47.6 %) | 6 (75.0 %) | 36 (45.0 %) | 150 (52.4 %) | 271 (50.1 %) | 127 (58.5 %) | 0.17 |
| Body mass index, kg/m² | 25.3 ± 5.1 | 27.1 ± 6.8 | 29.1 ± 4.0 | 26.3 ± 5.6 | 25.6 ± 5.9 | 25.2 ± 5.0 | 24.4 ± 4.7 | <0.001 |
| Medical history, N (%) |  |  |  |  |  |  |  |  |
| Hypertension | 10 (83.3 %) | 141 (74.6 %) | 4 (50.0 %) | 56 (70.0 %) | 136 (47.6 %) | 295 (54.5 %) | 87 (40.1 %) | <0.001 |
| Diabetes mellitus | 5 (41.7 %) | 71 (37.6 %) | 4 (50.0 %) | 29 (36.2 %) | 57 (19.9 %) | 106 (19.6 %) | 30 (13.8 %) | <0.001 |
| Coronary artery disease | 5 (41.7 %) | 36 (19.0 %) | 3 (37.5 %) | 19 (23.8 %) | 41 (14.3 %) | 46 (8.5 %) | 12 (5.5 %) | <0.001 |
| Atrial fibrillation | 5 (41.7 %) | 83 (43.9 %) | 4 (50.0 %) | 30 (37.5 %) | 48 (16.8 %) | 114 (21.1 %) | 29 (13.4 %) | <0.001 |
| Heart failure | 5 (41.7 %) | 86 (45.5 %) | 5 (62.5 %) | 42 (52.5 %) | 39 (13.6 %) | 61 (11.3 %) | 20 (9.2 %) | <0.001 |
| Prior HF admission, N (%) | 2 (16.7 %) | 37 (19.6 %) | 4 (50.0 %) | 22 (27.5 %) | 11 (3.8 %) | 13 (2.4 %) | 6 (2.8 %) | <0.001 |
| Medication, N (%) |  |  |  |  |  |  |  |  |
| ACEi/ARB | 7 (63.6 %) | 80 (43.7 %) | 3 (37.5 %) | 40 (51.3 %) | 85 (30.8 %) | 179 (34.8 %) | 50 (24.0 %) | <0.001 |
| Beta-blocker | 4 (36.4 %) | 63 (34.4 %) | 6 (75.0 %) | 34 (43.6 %) | 39 (14.1 %) | 123 (23.9 %) | 37 (17.8 %) | <0.001 |
| Diuretics | 3 (27.3 %) | 84 (45.9 %) | 4 (50.0 %) | 35 (44.9 %) | 80 (29.0 %) | 135 (26.2 %) | 27 (13.0 %) | <0.001 |
| Statin | 8 (72.7 %) | 55 (30.1 %) | 4 (50.0 %) | 24 (30.8 %) | 67 (24.3 %) | 108 (21.0 %) | 34 (16.3 %) | <0.001 |
| O2 flow, L/min (N=627) | 9.0 (3.0 - 12.0) | 4.0 (3.0 - 9.0) | 6.0 (3.0 - 9.0) | 6.0 (2.0 - 9.0) | 3.0 (2.0 - 6.0) | 4.0 (2.0 - 9.0) | 6.0 (2.5 - 9.0) | 0.063 |
| Physical examination, N (%) |  |  |  |  |  |  |  |  |
| Leg edema | 6 (50.0 %) | 108 (57.1 %) | 4 (50.0 %) | 45 (56.2 %) | 58 (20.3 %) | 86 (15.9 %) | 27 (12.4 %) | <0.001 |
| Jugular venous distension | 3 (27.3 %) | 13 (7.0 %) | 0 (0.0 %) | 7 (9.0 %) | 4 (1.4 %) | 11 (2.1 %) | 5 (2.3 %) | <0.001 |
| Rales | 6 (50.0 %) | 124 (67.8 %) | 5 (83.3 %) | 45 (59.2 %) | 75 (27.0 %) | 158 (30.4 %) | 41 (19.2 %) | <0.001 |
| Systolic BP, mmHg | 130.0 ± 26.8 | 139.1 ± 27.7 | 131.0 ± 26.3 | 138.3 ± 31.9 | 132.5 ± 23.6 | 128.3 ± 25.9 | 132.9 ± 24.0 | <0.001 |
| Heart rate, bpm | 104.8 ± 21.7 | 92.1 ± 20.6 | 101.1 ± 21.0 | 92.1 ± 23.2 | 96.8 ± 19.4 | 97.7 ± 20.6 | 92.9 ± 21.0 | 0.001 |
| Respiratory rate, /min | 26.3 ± 9.8 | 28.3 ± 8.2 | 23.2 ± 4.4 | 26.0 ± 7.2 | 25.8 ± 6.8 | 26.6 ± 8.3 | 24.7 ± 8.4 | 0.006 |
| Congestion score index | 2.2 ± 0.5 | 2.1 ± 0.6 | 2.5 ± 0.4 | 2.2 ± 0.6 | 1.1 ± 0.7 | 1.4 ± 0.7 | 0.9 ± 0.7 | <0.001 |
| Laboratory findings |  |  |  |  |  |  |  |  |
| Hemoglobin, g/dl | 13.2 ± 1.8 | 12.4 ± 1.8 | 12.7 ± 3.5 | 12.1 ± 2.0 | 13.5 ± 1.8 | 12.6 ± 2.0 | 13.0 ± 2.1 | <0.001 |
| White blood count | 1325  (11300 - 18005) | 12930  (9100 - 16470) | 17800  (10140 - 19540) | 11300  (7800 - 15180) | 11100  (8420 - 14300) | 11390  (8000 - 16200) | 10200 (8100 - 13600) | 0.005 |
| C-reactive protein, mg/dl | 3.5 (1.0 - 7.1) | 7.8 (2.6 - 13.8) | 2.0 (1.1 - 5.2) | 2.2 (1.0 - 6.5) | 3.0 (1.0 - 9.1) | 10.2 (4.3 - 17.6) | 2.4 (0.8 - 11.2) | <0.001 |
| Sodium, mmol/l | 136.9 ± 3.4 | 137.5 ± 4.3 | 138.1 ± 4.6 | 136.3 ± 5.8 | 136.6 ± 4.6 | 136.8 ± 6.1 | 137.2 ± 4.5 | 0.026 |
| Potassium, mmol/l | 4.2 ± 0.6 | 4.2 ± 0.7 | 4.3 ± 0.4 | 4.4 ± 0.6 | 4.2 ± 0.5 | 4.1 ± 0.6 | 4.1 ± 0.5 | <0.001 |
| Blood glucose, mmol/l | 12.6 ± 4.5 | 8.9 ± 3.9 | 12.4 ± 10.3 | 9.3 ± 4.5 | 7.0 ± 2.5 | 7.6 ± 3.3 | 7.0 ± 2.8 | <0.001 |
| BUN, mg/dl | 27.1 ± 12.1 | 30.3 ± 17.8 | 30.5 ± 15.7 | 33.9 ± 26.4 | 19.9 ± 14.3 | 26.7 ± 19.0 | 20.8 ± 12.1 | <0.001 |
| eGFR, ml/min/1.73m² | 71.0 ± 26.9 | 65.6 ± 28.5 | 57.8 ± 24.0 | 63.2 ± 36.8 | 91.9 ± 36.9 | 84.7 ± 74.6 | 89.0 ± 35.3 | <0.001 |

**4. They also concluded that the combination of congestion score index improved reclassification on top of the Brest score and BNP. However, the relationship between this index and prognosis should be presented.**

We are afraid that some of our wording had been misleading. Our paper is solely focused on diagnostic value of CSI. Indeed, our results showed that CSI improved reclassification of AHF diagnosis on top of the Brest score (which has been reported as a diagnostic score) plus BNP. Per this reviewer’s comment, we have modified the misleading sentences throughout our revised manuscript as below. We thank you for your helpful comment.

Methods

“The increase in discriminative value of the addition of CSI for AHF diagnosis on top of the aforementioned potential covariates were assessed using continuous net reclassification improvement (NRI).”

Results

“The addition of CSI on top of the Brest score significantly improved reclassification of AHF diagnosis [NRI (95%CI)=94.9 (83.5-106.2), p<0.001]”

“Furthermore, in patients with available BNP data (N=496), CSI still significantly improved reclassification of AHF diagnosis on top of BNP and the Brest score”

Discussion

“CSI significantly improved the reclassification of AHF diagnosis on top of the recognized clinical diagnostic markers of AHF and natriuretic peptides.”

“In the current study, CSI was found to improve reclassification of AHF diagnosis on top of the Brest score and BNP.”

**5. In Methods, precise diagnosis of study population is unclear. Furthermore, selection bias may be present because the admission criteria in this study population is undecided.**
Thank you for this important comment. In the PARADISE cohort, the hospital’s electronic charts (Resurgences®) were systematically reviewed by investigators to search for the records of all patients admitted for acute dyspnea in the emergency department; therefore, selection bias likely did not occur. Importantly, after excluding patients with shock status, all acute dyspneic patients were consecutively included in the current analysis. We have thus modified the manuscript to make it clear that we included consecutive patients, thus precluding the risk of selection bias, as below:

Methods

“The hospital’s electronic charts (Resurgences^®^) were systematically reviewed by investigators to search for the records of all patients admitted for acute dyspnea in the ED. All patients with signs or symptoms of dyspnea, or requiring oxygen therapy for dyspnea during (or prior to) their ED stay were included. Patients with shock or cardiac arrest were excluded from this analysis as dyspnea was not the primary condition triggering ED admission. The PARADISE cohort is consequently a cohort of unselected consecutive patients with acute dyspnea in the ED."

**6. In Methods, subjects receiving heart failure therapy and diagnosed "heart failure" at discharge during hospitalization due to other diseases were excluded?**
Thank you for this detailed feedback and important comment. We have made it clear in the revised version of our manuscript that patients with HF precipitated by other diseases (i.e. pneumonia) were not excluded. After excluding patients with initial shock and/or cardiac arrest, we did not exclude any patients depending on their discharge diagnosis.

Importantly, we focused on the discharge diagnosis as the main cause of acute dyspnea. Patients who had a history of HF may likely take HF therapy even if they are admitted for isolated pneumonia. Such patients were labeled as pneumonia since their main cause of acute dyspnea was considered as pneumonia. We have further added this specific comment in the revised version of the manuscript.

Methods

**“**Each physician had access to all ED medical charts as well as additional hospital admission test results and records (*e.g.*, echocardiography, natriuretic peptide level and patient response to diuretic/bronchodilator therapy) but were blinded to CSI quantification on CXR. Homogenous coding was ensured by a trained senior physician (TC). Importantly, to determine the discharge diagnosis, we focused on the main cause of acute dyspnea rather than background medical history or coexisting conditions.”

**7. Reference 1 (published in 2006) may not be reflected current practice.**

We have considered the following reference as ref 1. Thank you.

Introduction

“Acute heart failure (HF) is one of the leading causes of acute dyspnea in the emergency department (ED) [1] and is associated with a higher risk of morbidity and mortality.”

(reference)

1.	Mebazaa A, Yilmaz MB, Levy P, Ponikowski P, Peacock WF, Laribi S, et al. Recommendations on pre-hospital & early hospital management of acute heart failure: a consensus paper from the Heart Failure Association of the European Society of Cardiology, the European Society of Emergency Medicine and the Society of Academic Emergency Medicine. European Journal of Heart Failure. 2015;17(6):544-58. doi: 10.1002/ejhf.289.

**8. The author selected various factors as model 1 in logistic regression analysis. These factors were previously shown as prognostic factors?**

Thank you. We selected all confounders which were previously considered as a factor influencing HF diagnosis. Furthermore, our multivariable analyses included the Brest Score, which is a validated diagnostic score based on clinical parameters. We have modified the revised manuscript as below.

Methods

“Multivariable analyses included relevant confounders as previously shown [1]”

(reference)

1.	Chouihed T, Rossignol P, Bassand A, Duarte K, Kobayashi M, Jaeger D, et al. Diagnostic and prognostic value of plasma volume status at emergency department admission in dyspneic patients: results from the PARADISE cohort. Clin Res Cardiol. 2018. Epub 2018/10/30. doi: 10.1007/s00392-018-1388-y. PubMed PMID: 30370469.

**9. The authors described " an automated assessment of pulmonary congestion based on CSI could be developed・・・" in the discussion section. I may be a gap in their argument.**
We agree with this reviewer’s suggestion, and have removed this sentence from the revised version of our manuscript.

Clinical implications

~~“Alternatively, an automated assessment of pulmonary congestion based on CSI could be developed and subsequently implemented in current radiography software.”~~

**10. I wonder that a short time training of radiographic congestion score index is unconvincing.**
We agree with this reviewer’s comment. Although accurate and reproducible scoring was achieved after about 3-hour training period in the current study and previous study [1], this learning period may not always be acceptable and we need more evidence to answer this question. We have modified the limitations section per this comment. Thank you.

Limitations

“CSI is a semi-quantitative tool with some subjectivity. Although accurate and reproducible scoring was achieved after about 3-hour training period in the current study and one of our previous study [1], more evidence may be needed to ascertain the appropriate learning period.”

(reference)

1.	Kobayashi M, Bercker M, Huttin O, Pierre S, Sadoul N, Bozec E, et al. Chest X-ray quantification of admission lung congestion as a prognostic factor in patients admitted for worsening heart failure from the ICALOR cohort study. Int J Cardiol. 2019. Epub 2019/07/10. doi: 10.1016/j.ijcard.2019.06.062. PubMed PMID: 31281047.

**11. The authors described "the large-sized population sample, the relatively homogenous management and adjudication of diagnosis" in the discussion section, there is no basis for these statements.**

Per this reviewer’s comment, we removed this sentence from our manuscript.

**Reviewer #2:**

We thank this reviewer for his constructive suggestions.

The reviewers’ comments are stated in plain text **with bold letters**, the authors’ answers in plain text.

**I confine my remarks to statistical aspects of this paper. The basic approach is fine but I do have a few issues to resolve before I can recommend publication.

Abstract - What numbers come after the +- sign? (it seems like SD). Also, specify that the numbers in the parameter estimate are 95% CI**
Thank you for this comment. We have modified our abstract per this reviewer’s suggestion as follows:

Abstract

“Of 1,333 patients, mean (SD) age was 72.0 (18.5) years, 686 (51.5%) were men, and mean (SD) CSI was 1.42 (0.79).”

“CSI was significantly associated with acute HF diagnosis (adjusted-odds ratio for 0.1-unit CSI increase 1.19, 95%CI 1.16 to 1.22). The diagnostic accuracy of CSI for acute HF was excellent, whether alone [area under ROC curve (AUROC) 0.84, 95%CI 0.82 to 0.86] or in addition to clinical model (AUCROC 0.87, 95%CI 0.85 to 0.90). CSI improved diagnostic accuracy on top of clinical variables [Net reclassification improvement (NRI)=94.9%] and clinical variables plus BNP (NRI=55.0%). Good diagnostic accuracy was observed in the validation cohorts (AUROC 0.75, 95%CI 0.68 to 0.82).”

**p. 6 Don't use quartiles. Use the raw score and, if desired, evaluate nonlinearity with a spline. Categorizing a continuous variable is almost always a mistake. Frank Harrell in *Regression Modelling Stragies* says "nothing could be more disastrous".**
We agree with this reviewer’s suggestion, and we have removed Table 2 entitled “**Association of Radiographic Congestion Score Index with Discharge Diagnosis of Acute Heart Failure**” in the revised version of our manuscript. We now present spline curves for the association between CSI and AHF diagnosis in univariable and multivariable models including the Brest score in **Figure 2**. Thank you for this helpful addition to our manuscript.

Results

In this study, 289 (21.7%) patients were diagnosed with AHF at discharge. Higher CSI was significantly associated with AHF [OR (95%CI) for a 0.1-unit increase in CSI=1.22 (1.19-1.25), p<0.001] even after adjustment for potential clinical confounders [adjusted OR in CSI=1.18 (1.15-1.22), p<0.001] and the Brest score [adjusted OR in CSI=1.19 (1.16-1.22), p<0.001]. The association of CSI with AHF diagnosis using restricted cubic spline regression analysis is shown in **Figure 2**. CSI had a linear association with AHF diagnosis (p>0.05 for non-linearity), and higher CSI showed an increased risk of AHF diagnosis [OR for CSI score 1.0=4.09 (2.50 to 6.71) , OR for CSI score 1.5=14.30 (6.50 to 31.82) , OR for CSI score 2.0 =37.03 (16.09 to 85.26) – considering CSI score 0.5 as reference]. Similar results were observed after adjustment for the Brest Score (**Figure 2**).

**Figure 2. Association between Congestion Score Index and Discharge Diagnosis of Acute Heart Failure**

Multivariable model included the Brest score.

**Was collinearity evaluated?**

Correlation with Spearman test between CSI and clinical variables included in our logistic models is presented below. All correlation coefficients were less than 0.50, which suggest the absence of sizeable collinearity in our models.

|  | Spearman rho | p-value |
| --- | --- | --- |
| Brest score | 0.421 | <0.001 |
| BNP | 0.487 | <0.001 |
| Age | 0.479 | <0.001 |
| Body mass index | 0.122 | <0.001 |
| Hemoglobin | -0.239 | <0.001 |
| White blood cell | 0.089 | 0.001 |
| eGFR | -0.335 | <0.001 |

Per this reviewer’s suggestion, we have described these results in the Methods section of our revised manuscript as below.

Methods

“All correlation coefficients of variables included in the models were less than 0.50 with CSI, suggesting the absence of important in-model collinearity.”

**p. 7 Last para - this doesn't look gradual! Also, see comment above.**

We have removed this description. In addition, per your previous suggestions, the readers now have access to spline curve analysis for the association between CSI and AHF diagnosis.

Results

“The association of CSI with AHF diagnosis using restricted cubic spline regression analysis is shown in **Figure 2**. CSI had a linear association with AHF diagnosis (p>0.05 for non-linearity), and higher CSI showed an increased risk for AHF diagnosis [OR for CSI score 1.0=4.09 (2.50 to 6.71) , OR for CSI score 1.5=14.30 (6.50 to 31.82) , OR for CSI score 2.0 =37.03 (16.09 to 85.26) – considering CSI score 0.5 as reference]. Similar results were observed after adjustment for the Brest Score (**Figure 2**).”

**Reviewer #3:**

We thank this reviewer for his/her important and thoughtful comments.

The reviewers’ comments are stated in plain text **with bold letters**, the authors’ answers in plain text.

**In this retrospective, single-center study, the authors evaluated the diagnostic value of Congestion Score Index (CSI) using a semi-quantitative method on chest X-rays. The authors conclude that the CSI is capable of improving heart failure prediction models in those patients who come to the emergency department with the diagnosis of acute dyspnea and that are based on the combination of clinical data (Brest scale) and natriuretic peptides (BNP).**

**The usefulness of chest radiography to evaluate patients with acute dyspnea in the emergency department is evident, however, the latest guidelines for heart failure recommend caution in their interpretation, since up to 20% of heart failure patients will not present radiographic alterations. (Eur J Heart Fail. 2016 Aug;18(8):891-975. doi: 10.1002/ejhf.592.). In addition, lung ultrasound has demonstrated its superiority over chest radiography for the evaluation of acute dyspnea, showing a better sensitivity and specificity values. (Crit Care Resusc. 2016 Jun;18(2):124., JAMA Netw Open. 2019 Mar; 2(3): e190703.).
Despite being a retrospective study, it has a good sample size and the statistical methodology is well developed. Furthermore, the authors validate their results in another cohort, which supposes an added value. However, despite being well executed methodologically, I think the following points should be clarified before being accepted:**

**Comment 1: It is striking that only 10% of patients had a history of admission for heart failure. Furthermore, the percentage of cardiovascular diseases was relatively low despite the fact that the patients had an average age of 72 years. Do the authors believe that this fact could positively influence the results? In fact, in the group of patients with older age and in those who were more symptomatic (higher Brest scale), the AUC decreased the most.**

Thank you for this important comment. In the revised version of the manuscript, we have further showed in **Table 1** that 258 (19.4%) patients were previously diagnosed HF. In the present study, 21.7% of patients were diagnosed AHF at discharge, and this low proportion of AHF diagnosis may explain the relative low prevalence of cardiovascular diseases [i.e., prior history of HF, coronary artery disease (CAD) and atrial fibrillation (AF)]. Indeed, the prevalence of these cardiovascular diseases were significantly higher in patients with AHF diagnosis [prior HF, 138 (47.8 %) in AHF patients and 120 (11.5 %) in non-AHF patients, p<0.001; CAD, 63 (21.8 %) in AHF patients and 99 (9.5 %) in non-AHF patients, p<0.001; AF, 122 (42.2 %) in AHF patients and 191 (18.3 %) in non-AHF patients, p<0.001]. Importantly, in our center (as in most centers in France), some patients known to have HF and all patients with obvious evidence of myocardial ischemia were directly admitted in the intensive cardiac care unit/cardiology ward, not through the emergency department [1]. Therefore, this low proportion of AHF diagnosis possibly due to French health care system may influence our results. However, it should be acknowledged that, recently, hospitalization rates for worsening HF in the ED declined over past decades [2]. It may be consequence of the development of disease management program to prevent urgent HF hospitalization [3]. Importantly, the proportion of AHF diagnosis in the current study is consistent with that of dyspneic patients in contemporary data [4, 5]. However, we agree with this reviewer’s overall point and have considered as a limitation in the revised manuscript.

Limitations

“The overall proportion of AHF diagnosis was relatively low, which may explain the fact that a low number of patients had congestion signs (i.e., leg edema, rales and jugular venous distention) and cardiovascular diseases. In our center (as in most centers in France), some dyspneic patients known to have HF and all patients with obvious evidence of myocardial ischemia were admitted directly in the intensive cardiac care unit/cardiology ward, not through the ED [1]. The proportion of AHF diagnosis possibly due to the health care system may influence our results. However, it should be noted that hospitalization rates for worsening HF in the ED declined over past decades [2], which may result from the development of disease management program to prevent urgent HF hospitalization [3]. Indeed, the proportion of AHF diagnosis in the present study was similar to that of dyspneic patients in other contemporary cohorts [4, 5].”

(references)

1.	Logeart D, Isnard R, Resche-Rigon M, Seronde MF, de Groote P, Jondeau G, et al. Current aspects of the spectrum of acute heart failure syndromes in a real-life setting: the OFICA study. Eur J Heart Fail. 2013;15(4):465-76. Epub 2012/11/29. doi: 10.1093/eurjhf/hfs189. PubMed PMID: 23186936.

2.	Schmidt M, Ulrichsen SP, Pedersen L, Bøtker HE, Sørensen HT. Thirty-year trends in heart failure hospitalization and mortality rates and the prognostic impact of co-morbidity: a Danish nationwide cohort study. Eur J Heart Fail. 2016;18(5):490-9. Epub 2016/02/13. doi: 10.1002/ejhf.486. PubMed PMID: 26868921.

3.	Bradley EH, Curry L, Horwitz LI, Sipsma H, Wang Y, Walsh MN, et al. Hospital strategies associated with 30-day readmission rates for patients with heart failure. Circulation Cardiovascular quality and outcomes. 2013;6(4):444-50. Epub 2013/07/19. doi: 10.1161/circoutcomes.111.000101. PubMed PMID: 23861483; PubMed Central PMCID: PMCPMC3802532.

4.	Januzzi JL, Jr., Chen-Tournoux AA, Christenson RH, Doros G, Hollander JE, Levy PD, et al. N-Terminal Pro-B-Type Natriuretic Peptide in the Emergency Department: The ICON-RELOADED Study. J Am Coll Cardiol. 2018;71(11):1191-200. Epub 2018/03/17. doi: 10.1016/j.jacc.2018.01.021. PubMed PMID: 29544601.

5.	Ibrahim I, Kuan WS, Frampton C, Troughton R, Liew OW, Chong JP, et al. Superior performance of N-terminal pro brain natriuretic peptide for diagnosis of acute decompensated heart failure in an Asian compared with a Western setting. Eur J Heart Fail. 2017;19(2):209-17. Epub 2016/09/14. doi: 10.1002/ejhf.612. PubMed PMID: 27620387.

**Comment 2: If it is possible, it would be interesting to perform a sub-analysis of the ejection fraction (HFrEF vs. HFpEF). Patients with HFrEF usually debut with increased congestion and it would be interesting to see how CSI behaves in these subgroups.**

This reviewer raises a very interesting question. We agree that the different severity/distribution of radiographic pulmonary congestion across levels of ejection fraction may provide new pathophysiologic insight on pulmonary congestion. However, in the present study, ejection fraction was not recorded since this parameter is not a major determinant of decision-making for acute dyspneic management [1]. Nonetheless, we agree that the lack of this parameter is a limitation in the revised manuscript as below.

Limitations

“In the present study, ejection fraction was not recorded, thus we did not evaluate the diagnostic accuracy of CSI across levels of ejection fraction. This parameter, however, is usually not a major determinant of decision-making for acute dyspneic management in the ED [1, 2].”

(references)

1.	Mebazaa A, Yilmaz MB, Levy P, Ponikowski P, Peacock WF, Laribi S, et al. Recommendations on pre-hospital & early hospital management of acute heart failure: a consensus paper from the Heart Failure Association of the European Society of Cardiology, the European Society of Emergency Medicine and the Society of Academic Emergency Medicine. Eur J Heart Fail. 2015;17(6):544-58. Epub 2015/05/23. doi: 10.1002/ejhf.289. PubMed PMID: 25999021.

2.	Chouihed T, Manzo-Silberman S, Peschanski N, Charpentier S, Elbaz M, Savary D, et al. Management of suspected acute heart failure dyspnea in the emergency department: results from the French prospective multicenter DeFSSICA survey. Scandinavian journal of trauma, resuscitation and emergency medicine. 2016;24(1):112. Epub 2016/09/19. doi: 10.1186/s13049-016-0300-x. PubMed PMID: 27639971; PubMed Central PMCID: PMCPMC5026775.

**Comment 3: As the authors well emphasize in the limitations section, up to 38% of the X-rays were difficult to interpret, I think it is an important fact, what was the degree of variability in the interpretation of the CSI in this group? If it is significant it should be taken into account.**

We agree that this result needs to clarify for the readers. In the present study, as shown in Figure 2, the assessment of CSI was easy, moderate and difficult in 237 (17.8%), 594 (44.6%) and 502 (37.7%) patients, respectively. However, AUC of AHF diagnosis decreased with its difficulty but to a lesser extent that we would have expected [AUC (95%CI)=0.92 (0.86 to 0.97) in easy, =0.84 (0.80 to 0.88) in moderate, =0.80 (0.75 to 0.84) in difficult]. We, in addition, found no significant interaction between CSI and its difficulty on diagnostic accuracy for AHF (P _interaction_=0.13). We believe this is really reassuring. However, to fully answer your concern, we have expanded the limitations section of the revised manuscript to further include statistical details.

Limitations

“Lastly, the assessment of CSI was difficult in 502 (37.7%) of patients which may limit its applicability in routine clinical practice. However, the diagnostic value of CSI persisted in these patients (AUC=0.80, 0.75-0.84) and the difficulty in assessing CSI did not influence its diagnostic accuracy (P_interaction_=0.13).”

**Comment 4: In the results and discussion section, it should be clear that the use of natriuretic peptides is useful because of their high negative predictive value. The degree of congestion (in this case measured by the CSI) should not be compared with the BNP concentration figures, BNP is not a good biomarker of congestion, this fact could explain the lower AUC.**

Thank you for this reviewer’s thoughtfulness of our results. In recent clinical practice guidelines, the measurement of natriuretic peptide is recommended to rule out alternative causes of acute dyspnea; thus, elevated levels of natriuretic peptide do not automatically confirm AHF diagnosis [1]. However, accumulated evidences suggested high diagnostic accuracy of natriuretic peptide in dyspneic patients over two decades [2-5]. Therefore, to answer this reviewer’s suggestion, we tone down our manuscript regarding natriuretic peptides related results. Importantly, we have removed the results showing comparison of diagnostic accuracy between CSI and BNP in the abstract section and in the revised figures. Furthermore, we have emphasized that BNP is generally used to rule out alternative dyspneic causes and highlighted its limitations throughout our revised manuscript as below. We believe this will answer your concern.

Abstract

“~~the accuracy of CSI combined with clinical variables was similar to the accuracy of BNP combined with clinical variables (delta AUROC=-0.01).~~

Discussion section entitled “**Radiographic Congestion Score Index and Other Diagnostic Measurements: Clinical Parameters and Natriuretic Peptide**”

“In the latest guidelines, natriuretic peptide is recommended to rule out non-HF related causes of acute dyspnea, although accumulated data suggested the overall diagnostic accuracy of natriuretic peptide [2, 6-9]. However, multiple comorbidity burden (i.e. older age and poor renal function) may lead to diagnostic uncertainly of natriuretic peptides in a sizeable proportion of dyspneic patients [10].”

(references)

1.	Ponikowski P, Voors AA, Anker SD, Bueno H, Cleland JG, Coats AJ, et al. 2016 ESC Guidelines for the diagnosis and treatment of acute and chronic heart failure: The Task Force for the diagnosis and treatment of acute and chronic heart failure of the European Society of Cardiology (ESC). Developed with the special contribution of the Heart Failure Association (HFA) of the ESC. Eur J Heart Fail. 2016;18(8):891-975. doi: 10.1002/ejhf.592. PubMed PMID: 27207191.

2.	Januzzi JL, Jr., Chen-Tournoux AA, Christenson RH, Doros G, Hollander JE, Levy PD, et al. N-Terminal Pro-B-Type Natriuretic Peptide in the Emergency Department: The ICON-RELOADED Study. J Am Coll Cardiol. 2018;71(11):1191-200. Epub 2018/03/17. doi: 10.1016/j.jacc.2018.01.021. PubMed PMID: 29544601.

3.	Ibrahim I, Kuan WS, Frampton C, Troughton R, Liew OW, Chong JP, et al. Superior performance of N-terminal pro brain natriuretic peptide for diagnosis of acute decompensated heart failure in an Asian compared with a Western setting. Eur J Heart Fail. 2017;19(2):209-17. Epub 2016/09/14. doi: 10.1002/ejhf.612. PubMed PMID: 27620387.

4.	Maisel AS, Krishnaswamy P, Nowak RM, McCord J, Hollander JE, Duc P, et al. Rapid measurement of B-type natriuretic peptide in the emergency diagnosis of heart failure. N Engl J Med. 2002;347(3):161-7. Epub 2002/07/19. doi: 10.1056/NEJMoa020233. PubMed PMID: 12124404.

5.	Januzzi JL, Jr., Camargo CA, Anwaruddin S, Baggish AL, Chen AA, Krauser DG, et al. The N-terminal Pro-BNP investigation of dyspnea in the emergency department (PRIDE) study. Am J Cardiol. 2005;95(8):948-54. Epub 2005/04/12. doi: 10.1016/j.amjcard.2004.12.032. PubMed PMID: 15820160.

6.	Krauser DG, Chen AA, Tung R, Anwaruddin S, Baggish AL, Januzzi JL, Jr. Neither race nor gender influences the usefulness of amino-terminal pro-brain natriuretic peptide testing in dyspneic subjects: a ProBNP Investigation of Dyspnea in the Emergency Department (PRIDE) substudy. J Card Fail. 2006;12(6):452-7. Epub 2006/08/17. doi: 10.1016/j.cardfail.2006.04.005. PubMed PMID: 16911912.

7.	Anwaruddin S, Lloyd-Jones DM, Baggish A, Chen A, Krauser D, Tung R, et al. Renal function, congestive heart failure, and amino-terminal pro-brain natriuretic peptide measurement: results from the ProBNP Investigation of Dyspnea in the Emergency Department (PRIDE) Study. J Am Coll Cardiol. 2006;47(1):91-7. Epub 2006/01/03. doi: 10.1016/j.jacc.2005.08.051. PubMed PMID: 16386670.

8.	Bayes-Genis A, Lloyd-Jones DM, van Kimmenade RR, Lainchbury JG, Richards AM, Ordonez-Llanos J, et al. Effect of body mass index on diagnostic and prognostic usefulness of amino-terminal pro-brain natriuretic peptide in patients with acute dyspnea. Arch Intern Med. 2007;167(4):400-7. Epub 2007/02/28. doi: 10.1001/archinte.167.4.400. PubMed PMID: 17325303.

9.	Daniels LB, Maisel AS. Natriuretic peptides. J Am Coll Cardiol. 2007;50(25):2357-68. doi: 10.1016/j.jacc.2007.09.021. PubMed PMID: 18154959.

10.	Roberts E, Ludman AJ, Dworzynski K, Al-Mohammad A, Cowie MR, McMurray JJ, et al. The diagnostic accuracy of the natriuretic peptides in heart failure: systematic review and diagnostic meta-analysis in the acute care setting. BMJ (Clinical research ed). 2015;350:h910. Epub 2015/03/06. doi: 10.1136/bmj.h910. PubMed PMID: 25740799; PubMed Central PMCID: PMCPMC4353288.

**Comment 5: Given that the authors acknowledge having experience in the use of lung ultrasound, it would be advisable to compare the results of this study, based on chest radiography, with other studies where lung ultrasound has been tested to identify patients with heart failure upon arrival at the emergency room. I think that such a comparison would make the discussion more attractive.**

Thank you for this very interesting comment. We would have loved to have lung ultrasound data, and compare diagnostic value between lung ultrasound and CSI in the future. Of note, prior reports did not apply our approach to quantify congestion evaluated from chest radiography. We nonetheless agree that recent studies showed the clinical utility of lung ultrasound to diagnose AHF in dyspneic patients with high specificity and high sensitivity [1-3], and its diagnostic performance was better than that of CXR[4, 5] (using however the classical approach to CXR).

Clinical implications

“Recent studies showed the clinical utility of lung ultrasound to diagnose AHF in dyspneic patients with high specificity and high sensitivity [1-3], and its diagnostic accuracy was better than that of CXR[4, 5]. Of note, these previous studies did not include quantitative assessment of radiographic pulmonary congestion. In any event, based on the promising results herein, multi-center trials may be warranted to assess the impact of the implementation of CSI on AHF diagnosis in patients with acute dyspnea. Furthermore, further study to compare diagnostic value between CSI on chest radiography and lung ultrasound is worthy undertaking.”

(references)

1.	Martindale JL, Wakai A, Collins SP, Levy PD, Diercks D, Hiestand BC, et al. Diagnosing Acute Heart Failure in the Emergency Department: A Systematic Review and Meta-analysis. Acad Emerg Med. 2016;23(3):223-42. Epub 2016/02/26. doi: 10.1111/acem.12878. PubMed PMID: 26910112.

2.	Pivetta E, Goffi A, Lupia E, Tizzani M, Porrino G, Ferreri E, et al. Lung Ultrasound-Implemented Diagnosis of Acute Decompensated Heart Failure in the ED: A SIMEU Multicenter Study. Chest. 2015;148(1):202-10. Epub 2015/02/06. doi: 10.1378/chest.14-2608. PubMed PMID: 25654562.

3.	Buessler A, Chouihed T, Duarte K, Bassand A, Huot-Marchand M, Gottwalles Y, et al. Accuracy of several lung ultrasound methods for the diagnosis of acute heart failure in the emergency department: A multicenter prospective study. Chest. 2019. Epub 2019/08/06. doi: 10.1016/j.chest.2019.07.017. PubMed PMID: 31381880.

4.	Maw AM, Hassanin A, Ho PM, McInnes MDF, Moss A, Juarez-Colunga E, et al. Diagnostic Accuracy of Point-of-Care Lung Ultrasonography and Chest Radiography in Adults With Symptoms Suggestive of Acute Decompensated Heart Failure: A Systematic Review and Meta-analysis. JAMA network open. 2019;2(3):e190703-e. doi: 10.1001/jamanetworkopen.2019.0703.

5.	Pivetta E, Goffi A, Nazerian P, Castagno D, Tozzetti C, Tizzani P, et al. Lung ultrasound integrated with clinical assessment for the diagnosis of acute decompensated heart failure in the emergency department: a randomized controlled trial. Eur J Heart Fail. 2019;21(6):754-66. Epub 2019/01/29. doi: 10.1002/ejhf.1379. PubMed PMID: 30690825.

**Comment 6: Figure 3 tries to include the AUCs of the different models, but in my point of view it is difficult to interpret. Probably a simpler table with the different variables included in each model and its AUC would be easier to interpret.**

We agree that a lot of results included in Figure 4 might be cumbersome for the readers. Thank you for this constructive suggestion. We have modified our figure, adding a table with the results of delta AUC/NRI models. However, if deemed necessary by this reviewer and/or the editorial team, we are pleased to further modify this figure in order to maximize readability.

**Figure 4. Added values of CSI and BNP for the Diagnosis of Acute Heart Failure on Top of the BREST score in Patients with Available BNP (N=496)**
